# Simulated bat populations erode when exposed to climate change projections for western North America

**DOI:** 10.1371/journal.pone.0180693

**Published:** 2017-07-07

**Authors:** Mark A. Hayes, Rick A. Adams

**Affiliations:** School of Biological Sciences, University of Northern Colorado, Greeley, Colorado, United States of America; CSIRO, AUSTRALIA

## Abstract

Recent research has demonstrated that temperature and precipitation conditions correlate with successful reproduction in some insectivorous bat species that live in arid and semiarid regions, and that hot and dry conditions correlate with reduced lactation and reproductive output by females of some species. However, the potential long-term impacts of climate-induced reproductive declines on bat populations in western North America are not well understood. We combined results from long-term field monitoring and experiments in our study area with information on vital rates to develop stochastic age-structured population dynamics models and analyzed how simulated fringed myotis (*Myotis thysanodes*) populations changed under projected future climate conditions in our study area near Boulder, Colorado (Boulder Models) and throughout western North America (General Models). Each simulation consisted of an initial population of 2,000 females and an approximately stable age distribution at the beginning of the simulation. We allowed each population to be influenced by the mean annual temperature and annual precipitation for our study area and a generalized range-wide model projected through year 2086, for each of four carbon emission scenarios (representative concentration pathways RCP2.6, RCP4.5, RCP6.0, RCP8.5). Each population simulation was repeated 10,000 times. Of the 8 Boulder Model simulations, 1 increased (+29.10%), 3 stayed approximately stable (+2.45%, +0.05%, -0.03%), and 4 simulations decreased substantially (-44.10%, -44.70%, -44.95%, -78.85%). All General Model simulations for western North America decreased by >90% (-93.75%, -96.70%, -96.70%, -98.75%). These results suggest that a changing climate in western North America has the potential to quickly erode some forest bat populations including species of conservation concern, such as fringed myotis.

## Introduction

The potential impacts of a changing climate on bat populations is of increasing concern [1–4], in part because bats represent a large contribution to mammalian species diversity [5] and ecosystem processes [6], including important economic impacts to agricultural systems [7–9]. Climate change projections for North America suggest that future conditions will be warmer and drier in some parts of western North America, including ecoregions that are currently arid [10–12]. These projections also suggest that previously aberrant weather and hydrologic patterns, such as longer and more intense droughts and substantially prolonged reductions in surface water and stream-flows, will be more frequent [12–16]. The temperate zone insectivorous bat populations that occur in western North America may be impacted by these changes. These species tend to be long-lived (e.g., >5 years)[17], but reproduce at most once per year, and usually produce only one viable offspring per reproductive year [17,18]. Thus, stable and growing bat populations in western North America require that, during their reproductive years, females regularly produce pups that survive to adulthood [19–21].

During the reproductive season many bat species in western North America establish maternity roosts where congregations of reproductive females give birth to and raise young [22]. While pregnant and lactating, reproductive females in warm and arid regions require constant access to high quality surface water resources that tend to be within a short commuting distance from maternity roosts [23–27]. Pregnancy and lactation are known to place substantial water stress on females in warm and arid environments [28–33]. As a result, reproductive females often establish maternity roosts close to predictable water resources and visit these water resources more frequently than do non-reproductive females. For example, Adams and Hayes [34] showed that reproductive female fringed myotis (*Myotis thysanodes*) visited an artificial water source near a maternity roost much more frequently than did non-reproductive females of the same species. These behavior patterns underscore the notion that pregnant and lactating females require more frequent access to surface water resources than do females that are not reproductively active.

Long-term field studies suggest that in some parts of western North America the proportion of reproductively active females tends to be lower during hotter and drier years, during which historically-used maternity roost sites may be substantially warmer and the availability of surface water is reduced [25,27]. Adams [25] evaluated reproductive condition in female insectivorous bats using 13 years of data (1996–2008) from the Colorado Front Range, and found that reproductive success was lowest in years that were hotter and drier. For example, in 2007 and 2008—two of the driest years of the study—less than 60% of fringed myotis females and less than 50% of little brown bat (*Myotis lucifugus*) females of reproductive age were determined to be reproductively active. Such a high proportion of non-reproductive females is in stark contrast to other parts of North America that are not arid. In New Hampshire during a similar period (1993–2008), Frick et al. [35] estimated that on average 95% of adult female little brown bats were reproductively active, with the lowest rate of 87% during 2008. The substantially lower reproductive rates during drier years observed in western North America may be the result of behavioral and physiological adaptations to arid ecoregions. Adams [25] proposed that during periods of heat and water stress non-reproductive females and reproductive females that abandon young are released from being anchored to maternity roosts that increase evaporative water loss, or that are near water resources but suboptimal for non-reproductive female roosting. Thus, during the hottest and driest years when roosts may be hottest and water resources may be farther from suitable maternity roosts—and regularly visiting these water resources requires additional energy and adds to water stress—a substantial proportion of females may not produce viable offspring that survive to adulthood. In arid regions, this strategy may have positive influences on individual survival rates and thus improve reproductive output over a female’s remaining reproductive years. However, pronounced and prolonged decreases in the proportion of females that produce viable offspring over years and decades may put some populations at risk of extinction [27,36].

Population dynamics models have been used to evaluate the potential impacts of a changing climate on species of management and conservation concern. For example, population dynamics models have been used to evaluate the impacts of increasing sea surface temperatures on North Sea cod populations [37] and the impacts of a changing climate on long-lived shorebirds [38]. Population models and simulations can be used to make predictions about population viability under varying environmental scenarios and to suggest management interventions that can improve population performance [39–42]. We hypothesized that the link between climate conditions and the proportion of reproductively active female bats proposed by Adams [25] could have substantial impacts on the bat populations in our study area, and that these impacts could over decades substantially erode bat populations if the climate becomes warmer and drier. However, the magnitude of the impacts of a changing climate on bat populations in our study area and elsewhere in western North America was not clear to us. The objective of this study was to use numerical simulations to analyze the potential relationships between a changing climate and the dynamics of bat populations living in western North America. Here, we focus on fringed myotis, which is a species of substantial conservation concern in the United States and may be particularly susceptible to a warmer, drier climate in western North America [25,26,34,43]. This study is part of a broader research program investigating the impacts of changing climate conditions on bat behavior and populations in the Southern Rocky Mountains, and elsewhere in western North America. A key goal of this program is to combine field experiments, collection and analysis of long-term datasets, and mathematical simulations to better understand potential impacts of a changing climate on bat populations in our study area, and develop insights into the potential impacts of a changing climate on bat populations in other parts of western North America.

We used age-structured, stochastic population dynamics models and simulations to explore the dynamics of hypothetical bat populations through the later portion of the 21^st^ century (the 2080’s), and incorporated into these population models the potential impacts of a changing climate on female reproductive success [25], based on downscaled global circulation models. We developed an age-structured population model for fringed myotis that incorporated plausible survival and fertility rates and that resulted in an approximately stable age distribution and a population growth rate (λ) of λ ≈ 1.0. We then explored the potential influence of a changing climate on fringed myotis populations in our study area, as well as generalized fringed myotis populations in western North America, using Monte Carlo simulations [39,43]. We view these fringed myotis simulations as “first approximation models” [44] and an initial phase of an ongoing evaluation of the risks that regional climate change pose to bat populations in western North America.

## Materials and methods

**Fig 1:**
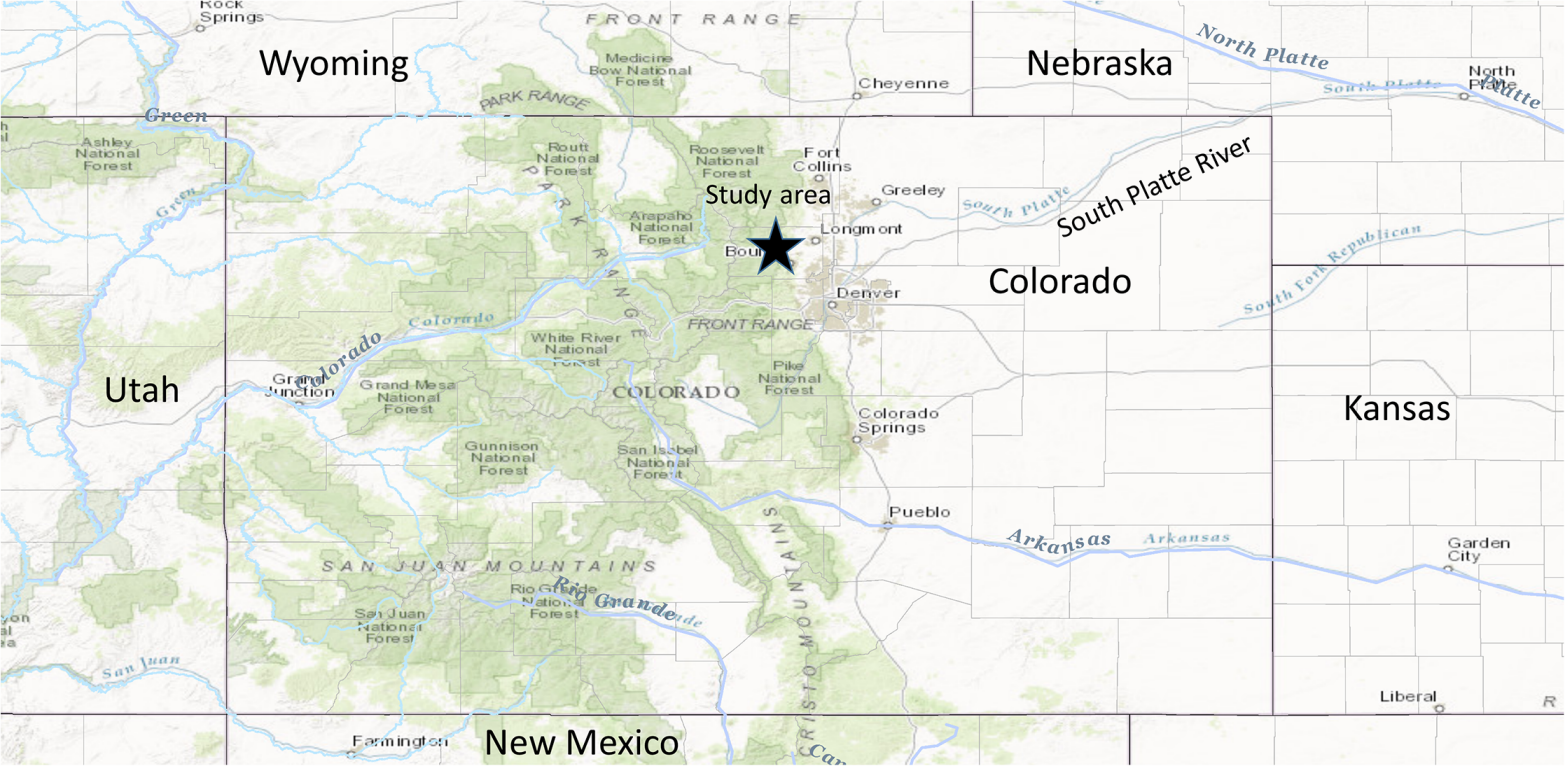
A map of Colorado indicating the location of the study area. The black star indicates the approximate location of the study area in Boulder County, Colorado.

We used population models to simulate and analyze the potential relationships between a changing climate and the population dynamics of fringed myotis (*Myotis thysanodes*) populations living in forested landscapes of Colorado’s Front Range and elsewhere in western North America. We explored the dynamics of simulated populations through year 2086 following standard procedures for developing deterministic and stochastic age structured population dynamics models [39–42], and by incorporating into these models the changes in climate projected by global circulation models under varying carbon emission scenarios downscaled to the approximate location of our study area in the Southern Rocky Mountains (Boulder County, Colorado; Fig 1). In this section we describe: how we built our age-structured population dynamics model, including how we parameterized vital rates; how we evaluated the sensitivity and elasticity of this model; how stochasticity was added to the model; how we developed a statistical model to predict female reproductive rates in future years using future climate projections; how we performed Monte Carlo simulations under various future climate projections; and finally the software used to perform the analysis.

**Fig 2:**
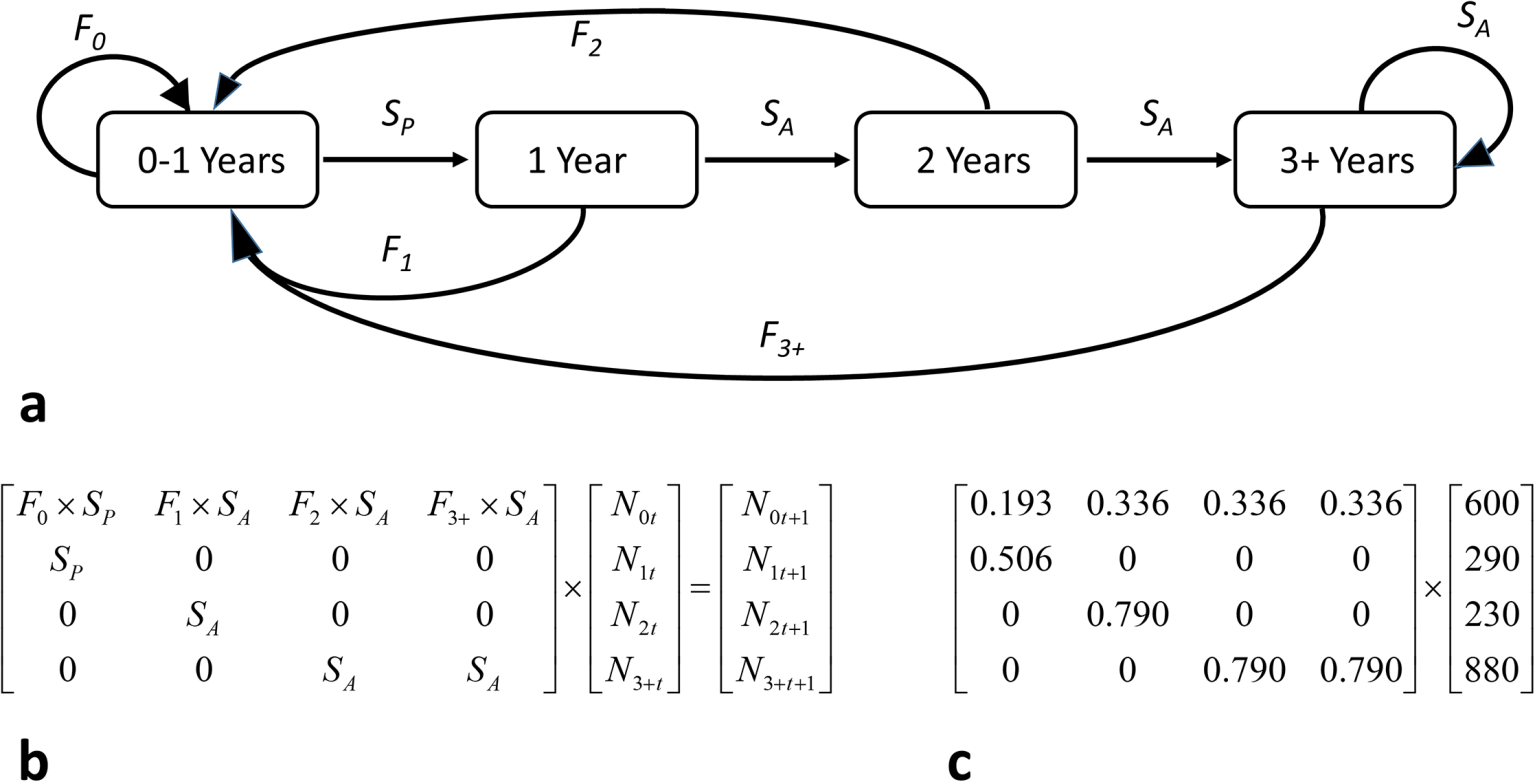
**Transition diagram (a), associated age-structured matrix model (b), and transition probabilities (c) used for the stable age-structured matrix model used in this analysis.** In the transition diagram (a), 4 stages are shown: bats that are 0–1 years old (0–1 Years); bats that are 1 to 2 years old (1 Year); bats that are 2 to 3 years old (2 Years); and bats that are 3 years or older (3+ Years). Each right-pointing arrow represents a possible transition in stage from one year to the next. On their third birthday, individual bats move from the 2 Years group to the 3+ Years group, and in all subsequent years while alive the bat stays in the 3+ Years group. *S*_*P*_ is the probability of a newborn pup surviving until its first birthday. Once bats are 1 year old, they are considered adults and *S*_*A*_ is the probability that the bat survives until its next birthday. *F*_*0*,_ *F*_*1*,_ *F*_*2*,_ and *F*_*3+*_ are fertility rates for each adult age class and represent the probability of an adult female bat in each age class giving birth to a viable pup. In the age-structured matrix model (**b**), *N*_*0t*_, *N*_*1t*_ *N*_*2t*_, and *N*_*3+t*_ are the number of bats in each class in year *t*, and likewise *N*_*0t+1*_, *N*_*1t+1*_ *N*_*2t+1*_, and *N*_*3+t+1*_ are the number of bats in each age class in the next year, *t+1*. Transition probabilities are shown in (**c**), along with the approximately stable age distribution for the initial population of 2,000 females.

We used what we believe to be plausible survival and fertility rates for forest bats in western North America, based on the literature and data from our study area [17,20,35,43,45]. Since we do not yet have capture-mark-recapture based estimates (e.g., [17,45]) for vital rates over our full study area, we relied on estimates from the literature to parameterize our models. We defined mean annual survival rate for adults (*S*_*A*_) as 0.79 following [17,45] for similar western forest species. For mean pup survival from birth to first birthday (*S*_*P*_) we used 0.64 [17,45]. To parameterize the mean fertility rate for a given age class (*F*_*i*_) we assumed that the age distribution at birth was equal (e.g., a 1:1 female:male birth ratio), that the mean litter size was 1.0, and that these values remained stable throughout all simulations. We assumed that the mean fertility rate for adult females (*F*_*A*_) was 0.85 under stable conditions, and that the mean fertility rate for yearling females (*F*_*0*_) was 90% of the adult fertility rate. The adult fertility rate of 0.85 is almost identical to the estimated mean fertility rate for 1996–2008 estimated for our study area by our logistic regression model for fertility using all available data from our study area (0.8496; see below). We treated birth as a discrete process that occurs on the last day of a given simulated year such that the proportion of females in a given age class that give birth is a function of the probability of surviving the year and the probability of being reproductively active that year (e.g., *S*_i_*F_*i*_). We counted the number of individuals in each age class on the first day of each simulated year. Given these assumptions, the expected values (E) of female offspring (*O*) produced per reproductive female are: E[*O*_*A*_] = (0.5)(0.85) = 0.425 and E[*O*_*Y*_] = (0.50)(0.85)(0.90) = 0.3825. These vital rates are similar to those reported by O'Shea et al. ([45]; a 3-class model) for big brown bats (*Eptesicus fuscus*) in maternity roosts associated with buildings and forests in and near Fort Collins, Colorado, and are similar in structure to the generalized model of Indiana bats (*Myotis sodalis*) use by Hallam and Federico ([20]; a 15-class model). The vital rates we used were intended to be both plausible and result in an approximately stable 4-stage matrix model when analyzed analytically and when projected through an approximately stable Monte Carlo simulation (see below). In all simulations, we used an initial population of 2,000 females as follows: 600 pups; 290 1-year-old females; 230 2-year-old females; and 880 3-year-and-older females. We followed the approach of previous researchers in only considering females, given that stable female populations are critical to maintaining stable populations of sensitive species [39–42]. The transition diagram used to model age-structured dynamics of the population (a), associated matrix model (b), and transition probabilities (c) are shown in Fig 2.

This age-structured population model for fringed myotis was designed to result in an approximately stable initial age distribution and an asymptotic population growth rate of λ ≈ 1.0. We do not currently know if fringed myotis populations are stable, growing, or decreasing, but for the purposes of this analysis we assume that the simulated fringed myotis populations were stable during the recent past (e.g., 1950–2000). The initial age distribution was close to a stable age-distribution of approximately two thousand females. The dominant eigenvalue (λ) and eigenvectors (***w***) were calculated to determine the population growth rate and stable age distributions of the population. We assumed that female bats gave birth to either 1 or 0 pups per year, and therefore fertility rates were constrained between 0 and 1 and expressed as females born per year per adult female. It is possible that forest-dwelling *Myotis* occasionally give birth to twins [45], but for this analysis we assumed that only single pups were born, survived parturition, and become successfully volant. In this situation, all vital rates are constrained between 0 and 1. We conducted sensitivity analysis for the matrix elements [39,40,42] by individually varying estimated fertility and survival rates by minus ten percent (-10%; e.g. rate × 0.90), while keeping other vital rates at their original value. This sensitivity analysis describes the relative effects of changes in fertility and survival rates on the population growth rate (λ). We then evaluated elasticity, which estimates the effects of proportional changes in vital rates on λ. Elasticity of a given matrix element (*e*_*ij*_) follows [42] and is:

$$e_{ij}=\frac{a_{ij}}{\lambda }\frac{\partial \lambda }{\partial a_{ij}},$$

where *a*_*ij*_ is the value of a given matrix element, and ∂*λ* is the change in λ given a change in the value of the matrix element, ∂*a*_*ij*_. This approach allows elasticities to be directly compared among vital rates [39,42].

We added demographic stochasticity to all models for each age class, year, vital rate, and simulation run by incorporating a pseudo-random number generator into vital rates using a triangular probability density function. This function randomly selected a vital rate between the minimum and maximum specified vital rate, with the peak of the distribution positioned at the median vital rate. Using a triangular distribution produces relatively heavy tails but keeps the vital rates constrained between the minimum and maximum values. We also introduced environmental stochasticity into fertility rate estimates. For our Boulder County study area (Boulder Models), we used the minimum and maximum fertility rates predicted using a logistic regression model (see below) and minimum and maximum temperature and precipitation projections for a given future year. We developed range-wide fringed myotis models (General Models), using information from occurrence location records for fringed myotis available in the Global Biodiversity Information Facility database (www.gbif.org) and future climate projections from 11 future climate models (details below; Table 1). We drew adult fertility rates from a triangular distribution with minimum and maximum of 0.90 and 1.10 multiplied by the prediction for each age class. Thus, if predicted fertility for a given year and age class is high, the mean number of simulated offspring produced by the age class could exceed 1.0. This environmental stochasticity was used in Monte Carlo simulations in the same way as above for demographic stochasticity, by incorporating a pseudo-random number generator into fertility rates using a triangular probability density function. This function randomly selected a fertility rate between the minimum and maximum predicted for a given year and age class, with the peak of the distribution positioned at the median fertility rate.

**Table 1:** List of the CMIP5 future climate models used in this study. The CMIP5 climate models are the World Research Programme’s most current Coupled Model Intercomparison Project set of future climate projections for year 2070 (CMIP5; cmip-pcmdi.llnl.gov/cmip5/). For each model the four representative concentration pathways (RCP2.6, RCP4.5, RCP6.0, RCP8.5) associated with the model were used.

Model Name	Abbreviation	Nation	Institution
BCC-CSM1-1	BC	China	Beijing Normal University
CCSM4 (NCAR-UCAR)	CC	USA	National Center for Atmospheric Research
GISS-E2-R	GS	USA	NASA/Goddard Institute for Space Studies
HadGEM2-AO	HD	UK	Met Office Hadley Centre
HadGEM2-ES	HE	UK	Met Office Hadley Centre
IPSL-CM5A-LR	IP	France	Institut Pierre Simon LaPlace
MIROC5	MC	Japan	Japan Agency for Marine Earth Science
MIROC-ESM-CHEM	MI	Japan	Japan Agency for Marine Earth Science
MIROC-ESM	MR	Japan	Japan Agency for Marine Earth Science
MRI-CGCMM3	MG	Japan	Meteorological Research Institute
NorESM1-M	NO	Norway	Norwegian Climate Centre

To build a correlative statistical model of the influence of climate on adult fertility (*F*_*i*_; the proportion of individuals that are reproductively active in a given age class during a given year), we fit a binary logistic regression model using mean annual temperature and annual precipitation for each year from 1996–2008 to Hayes’ female reproductive success data for fringed myotis in Boulder County, Colorado [43], using U.S. National Oceanic & Atmospheric Administration (NOAA) Earth System Research Laboratory mean annual temperature and annual precipitation data for Boulder, Colorado (www.esrl.noaa.gov/psd/). In this model we used the annual mean temperature (°C) for a given year using the NOAA data from the same reference period (1986–2005) as used by the NCAR-UCAR CCSM4 future climate models (Table 1; https://gisclimatechange.ucar.edu/). The mean annual temperature for this reference period for Boulder, Colorado was 10.85°C. We likewise used the annual precipitation for a given year (reference period mean = 541 mm), also using the NOAA data. We fit a binary logistic response function [46] for the female reproductive data from our study area:

$$\mathrm{Pr}\{Y=1\}=\frac{e^{\beta_{0}+\beta_{T}(T)+\beta_{P}(P)}}{1+e^{\beta_{0}+\beta_{T}(T)+\beta_{P}(P)}},$$

where Pr{*Y* = 1} is the predicted mean probability of adult females in a given age class being reproductively active given the temperature (*T*) and precipitation (*P*) conditions for a given year, and the *β*_*i*_’s are beta estimates for the logit model. We considered both annual temperature and annual precipitation to be highly plausible abiotic covariates, which were not highly correlated [46]; on the other hand, we recognized that the factors influencing fertility for a given age class likely involve a complex suite of abiotic and biotic factors that are not likely to be fully captured by this simplifying model, and that no future climate model is yet capable of capturing these covariates. Thus, we emphasize that the logistic model used here and the simulation results presented are only used as first approximation models [44] to evaluate the conjecture that a changing climate might have non-trivial impacts on some simulated bat populations. To build a dataset for the logistic fertility model, we used the data related to adult female fringed myotis captured or collected in Boulder County, Colorado, for 1996–2008 compiled by Hayes (see [43] for a full list). We used data in which the biologist or collector listed the following information: the species; that the sex of the individual was female; that the individual was an adult; and that the reproductive status of the adult female individual was evaluated and determined as non-reproductive, pregnant, lactating, or post-lactating; if the reproductive status of the bat was listed as unknown or not listed, the information from that record was not used in the analysis. Early in the reproductive season (for example in May and early June) it can be difficult to identify whether individuals are pregnant. Similarly, it can be difficult to identify if a female is post-lactating in the late summer. Therefore, we restricted the data set to those records collected by a biologist or collector who we expected had the knowledge, skills, and experience to accurately identify the species, sex, age, and reproductive condition of individual bats in the Southern Rockies. The Boulder study area for this analysis was within the Southern Rockies ecoregion and includes foothills shrublands and mid-elevation forests, and is within the South Platte River basin.

We developed and ran 13 Monte Carlo simulations for fringed myotis populations in western North America using the age-structured population model as follows. We used a Stable Model, which assumed that vital rates in our study area in Boulder County, Colorado, will stay approximately at or near initial values through year 2086. We used 4 Boulder Models with yearly mean annual temperature and annual precipitation values projected by each of the 4 emission scenarios (RCP2.6, RCP4.5, RCP6.0, RCP8.5) using the NCAR-UCAR future climate models for each year from 2009–2086. The RCP2.6 emissions scenario assumes the least change in carbon emissions from historic levels and the RCP8.5 scenario assumes the largest increase in carbon emissions. We also used 4 Boulder Ensemble Models with averaged projections for mean annual temperature and annual precipitation values for year 2070 derived from the mean projection of 11 future climate models (Table 1); we used the year 2070 climate values to estimate fertility rates for this year and then projected values for each year between 2009–2069 and 2071–2086 for each of the 4 emission scenarios using a step function such that each year including 2070 represented an equivalent change in adult fertility. Finally, we used 4 General Models with projected mean annual temperature and annual precipitation values for year 2070 for each of the >2,000 fringed myotis occurrence locations in the Global Biodiversity Information Facility (www.gbif.org) dataset, and with the mean fertility rate prediction for all locations in 2070 using the same logistic model as used for the Boulder Models; for each year between 2009–2069 and 2071–2086 we calculated fertility rates using a step function such that each year including 2070 represented an equivalent change in adult fertility in the same way as for the Boulder Ensemble Models. In this analysis we focused on the influence of fertility rates (*F*_*i*_) on population dynamics and assumed that survival rates were not substantially affected by climate conditions, and the sex ratio of newborns was at parity in all years. These are simplifying assumptions for this analysis. To calculate fertility rates for the General Models, for each occurrence location we extracted the mean annual temperature and annual precipitation projection for years 1950–2000. We then used the temperature and precipitation projections for each location for year 2070 using each of the 11 future climate models, and used these projections to calculate a grand ensemble mean fertility projection for all locations (future climate data available at www.worldclim.org; 10 minute data). By combining climate information for pooled range-wide occurrence locations, the General Models represent ensemble simulations of the potential influence of changing climate conditions on a hypothetical fringed myotis population in western North America, whose mean fertility rates are equal to the mean fertility rates for the occurrence records in the GBIF dataset. Each Monte Carlo simulation consisted of 10,000 runs of 77 years representing years 2009 to 2086. For each Monte Carlo simulation we calculated the mean of the 10,000 final populations in year 2086, the percent change in the mean population from the original 2,000 females (% change), the minimum population size, the maximum population size, and the standard deviation of the populations. As a final step we created maps of fringed myotis occurrence locations in western North America and mapped the change (Δ_*i*_) in estimated fertility rate (*F*_*i*_) for each cell (with each cell 10 minutes of a degree) using future climate projections derived from the General Ensemble Model projections for year 2070 using the four representative concentration pathways (RCP2.6, RCP4.5, RCP6.0, RCP8.5). We considered using a finer-scale resolution, such as ~1 km^2^, but concluded that the courser resolution used here reflects a scale that is closer to the home range of a fringed myotis [43]. We also concluded that this resolution is appropriate for considering the influences of a changing climate on the landscapes used by individual bats over the course of a year. The map indicates the mean predicted change in fertility derived from the 11 future climate models when comparing mean values for 1950–2000 climate to year 2070 projections for the ensemble models. We also calculated mean change ($\bar{\Delta }$) in fertility rates for all fringed myotis locations used.

Wolfram Mathematica^®^ 10 (Wolfram Research, Inc., Champaign, Illinois) software was used to perform linear algebra calculations, sensitivity and elasticity analysis, and Monte Carlo simulations. Logistic regression analysis was conducted using the logit link function and Stata statistical software (StatCorp, College Station, Texas). ArcMap 10.3 (ESRI, Redlands, CA) software was used for climate data extraction, raster algebra, and map creation. A data and code package is available in a GitHub repository (https://github.com/mark-a-hayes/Hayes-Adams-2017).

## Results

The proportion of 190 adult female fringed myotis (*Myotis thysanodes*) that were reproductively active in 1996–2008 ranged from 0.727–1.000 (ave = 0.887; 1996 = 0.818, 1997 = 1.000, 1998 = 0.846, 1999 = 0.957, 2000 = 0.846, 2001 = 1.000, 2002 = 1.000, 2003 = 0.900, 2004 = 0.944, 2005 = 0.813, 2006 = 0.805, 2007 = 0.727, 2008 = 0.778). Sensitivity and elasticity results suggest that survival of older females has the most influence on population growth rates (Table 2). Elasticity was lowest for fertility associated with younger females (*F*_*0*_, *F*_*1*_, *F*_*2*_), and highest for survival rates, especially the three-year-old-and-older age class (*S*_*3+*_) (Table 2). The logistic regression analysis using female reproductive success data from 1996–2008 resulted in the following model:

$$\mathrm{Pr}\{Y=1\}=\frac{e^{\beta_{0}+\beta_{T}(T)+\beta_{P}(P)}}{1+e^{\beta_{0}+\beta_{T}(T)+\beta_{P}(P)}}=\frac{e^{2.6419-0.2534(T)+0.0040(P)}}{1+e^{2.6419-0.2534(T)+0.0040(P)}},$$

where *T* is mean annual temperature (°C) and *P* is the total precipitation (mm) for a given year. The NCAR-UCAR future climate models project that the temperature in our study area will increase over historic values (https://gisclimatechange.ucar.edu/inspector; place name = “Boulder, Boulder County, Colorado”), with the lower emission scenario (RCP2.6) predicting relatively stable mean annual temperature increase of ~1.2°C after about year 2020, but with the other emission scenarios (RCP4.5, RCP6.0, and RCP8.5) all predicting increases in temperature through 2086 (e.g., > 4.0°C increase for the RCP8.5 scenario). However, the NCAR-UCAR future climate models (and other future climate models and the Ensemble Model) do not predict substantially drier conditions in terms of annual precipitation in our study area (e.g. the NCAR-UCAR model: https://gisclimatechange.ucar.edu/inspector; place name = “Boulder, Colorado”).

**Table 2:** Sensitivity and elasticity of matrix elements to a -10% change in each vital rate used in the original matrix model. *F*_*0*,_ *F*_*1*,_ *F*_*2*,_ and *F*_*3+*_ are fertility rates for each adult age class. *S*_*0*,_ *S*_*1*_, *S*_*2*_, *S*_*3+*_ are the probabilities of an individual surviving until its next birthday.

Parameter	Sensitivity	Elasticity
*F*_*0*_	0.208	0.040
*F*_*1*_	0.103	0.034
*F*_*2*_	0.082	0.027
*F*_*3+*_	0.318	0.107
*S*_*0*_	0.333	0.168
*S*_*1*_	0.171	0.135
*S*_*2*_	0.136	0.107
*S*_*3+*_	0.459	0.362

**Fig 3:**
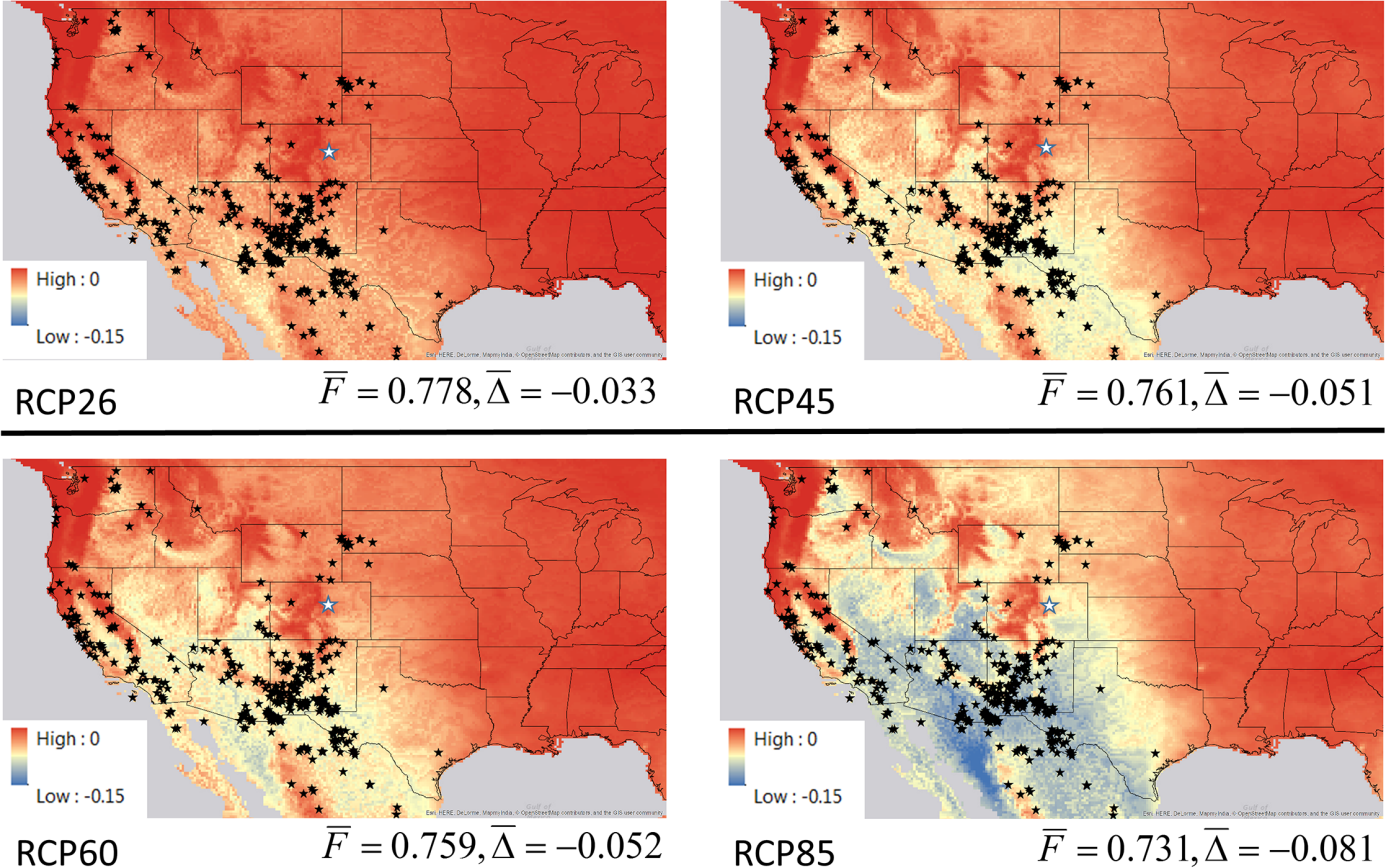
Maps of fringed myotis (*Myotis thysanodes*) occurrence locations in western North America and projected change in adult female fertility rates using future climate projections derived from the General Ensemble Model for year 2070 using the four representative concentration pathways (RCP2.6, RCP4.5, RCP6.0, RCP8.5). Fringed myotis locations are indicated by black stars, and our study area near Boulder, Colorado is indicated by a white star. The color ramp indicates the change (Δ_*i*_) in estimated fertility rate for a given cell on the map, using the estimated mean derived from the 11 future climate models when comparing 1950–2000 climate to year 2070 projections for the ensemble model. Darkest red indicates no change between 1950–2000 climate and future climate projections, and darkest blue indicates greatest negative change. Mean projected adult fertility rates for the fringed myotis locations ($\bar{F}$) and the mean change ($\bar{\Delta }$) from current climate conditions are also shown.

The Monte Carlo simulation results for the Stable Population Model, the Boulder Models, and the range-wide General Models are shown in Table 3. These results show the results from coupling the future climate projections with the logistic predictions for fertility through year 2086. Maps of 2,038 fringed myotis occurrence locations in western North America derived from the GBIF dataset (www.gbif.org) and projected changes in adult female reproductive rates (Δ_*i*_) using future climate projections derived from the General Ensemble Model projections for year 2070 and the four representative concentration pathways (RCP2.6, RCP4.5, RCP6.0, RCP8.5) are shown in Fig 3. The change in estimated fertility rate (*F*_*i*_) for a given cell on the map (Δ_*i*_), uses the estimated mean derived from the 11 future climate models when comparing 1950–2000 climate to year 2070 projections for the ensemble model.

**Table 3:** Results of Monte Carlo simulations of bat populations using an initial population of 2,000 females, the demographic information derived from our study area in Boulder County, Colorado, and future climate projections derived from the NCAR-UCAR Community Climate System (CCSM4) and General Ensemble Model projections for our study area (Boulder models) and western North America (General models) through year 2086. Mean, minimum, maximum, and standard deviation for final populations in year 2086 using 10,000 runs are shown for each scenario. Four emission trajectories were used (RCP2.6, RCP4.5, RCP6.0, RCP8.5), where the RCP2.6 scenario assumes the least change in emissions from historic levels and the RCP8.5 scenario assumes the largest change in carbon emissions.

Monte Carlo Simulation	Mean	% Change	Minimum	Maximum	Standard Deviation
Stable Population	2,070	+3.50	944	3974	415
Boulder RCP2.6 Ensemble	2,049	+2.45	862	5,535	418
Boulder RCP4.5 Ensemble	1,118	-44.10	512	2,413	229
Boulder RCP6.0 Ensemble	1,101	-44.95	517	2,594	226
Boulder RCP8.5 Ensemble	423	-78.85	153	956	88
Boulder RCP2.6 NCAR Model	2,582	29.10	1,248	5,376	519
Boulder RCP4.5 NCAR Model	2,001	+0.05	783	4,392	408
Boulder RCP6.0 NCAR Model	1,931	-0.03	898	4,178	390
Boulder RCP8.5 NCAR Model	1,106	-44.70	530	2,422	231
General RCP2.6 Model	125	-93.75	61	267	26
General RCP4.5 Model	66	-96.70	29	144	14
General RCP6.0 Model	66	-96.70	28	148	14
General RCP8.5 Model	25	-98.75	10	70	5

## Discussion

In this analysis fringed myotis populations increased or remained stable in 4 simulations for our Boulder study area (RCP2.6 Ensemble, RCP2.6 NCAR, RCP4.5 NCAR, RCP6.0 NCAR models), and decreased substantially in 4 of the simulations (RCP 4.5 Ensemble, RCP 6.0 Ensemble, RCP 8.5 Ensemble, and RCP 8.5 NCAR models). All of the range-wide General Models eroded substantially over the 77 year simulations, with all representative concentration pathways (RCP2.6, RCP4.5, RCP6.0, RCP8.5) resulting in >90% reduction in simulated fringed myotis populations (Table 3). This result suggests that fringed myotis populations in some parts of western North America have the potential to be substantially impacted by a changing climate. Our results suggest that fringed myotis populations may be particularly susceptible to the impacts of a changing climate in the southwestern U.S. (e.g., Arizona and New Mexico) and in northern México (Fig 3). These results support the hypothesis that regional climate change and related ecosystem changes have the potential to result in significant reductions in some forest bat populations in the Southern Rocky Mountains [25,34,43] and other arid regions in North America and elsewhere [27,47]. These results also support the hypothesis that a changing climate in western North America has the potential, by gradually eroding some populations, to radically change the geographic distributions of some bat species, reorder patterns of species richness and diversity, and alter the ecosystem services provided by bats. It is possible that fringed myotis and other forest bat populations might adapt to changing climate conditions either through behavioral adaptations (such as moving to more suitable locations and/or selecting different roosting sites) or via phenotypic plasticity. Investigation of behavioral and/or phenotypic adaptations to a changing climate could be fruitful directions for future research. However, our results suggest that even relatively small, but persistent, reductions in fertility rates can result in eroding populations. Sensitivity and elasticity results suggest that conservation strategies aimed at improving and maintaining fertility rates, as well as those aimed at supporting survival rates, might be best directed at mature females, such as those three years and older (Table 2).

The impacts of a changing climate in North America are projected to result in warmer and drier conditions in the southwestern U.S. where most future climate models project increasing annual mean temperatures and decreasing annual precipitation [13,48]. Although all CMIP5 future climate models project increasing temperatures in Colorado, there is less certainty about the impacts on annual precipitation in Colorado [48]. The southwestern U.S. is projected to become substantially drier, while northern portions of the continent are projected to become wetter, with Colorado in an area between these regions [48]. Generally southern Colorado is projected to become drier than northern Colorado, with Boulder County, which is in the northern half of Colorado, currently being projected to become substantially warmer, potentially without a substantial change in mean annual precipitation. The modeling approach employed here of coupling local climate change projections with a statistical model of female fertility rates and bat population dynamics simulations may be useful in analyzing the impacts of regional climate and ecological change on species of conservation concern in other ecoregions. This approach may also be useful in helping to prioritize bat species for limited conservation and management resources. For example, our results suggest that fringed myotis populations in the southwestern U.S. (e.g., New Mexico and Arizona) and in northern México may be at relatively more risk from the influences of a changing climate than populations in more northern parts of this species distribution (e.g., the Pacific Northwest, Wyoming, and South Dakota) (Fig 3). Future monitoring, field work, and analysis could be directed at areas of the western United States that are projected to become both warmer and substantially drier [48]. In our study area, fringed myotis and other forest bat species often use ephemeral water resources for drinking water after emerging from day roosts and during nightly foraging bouts, and maternity roost sites appear to be anchored to predictable and high quality resources [24–26,34,43,51–53]. These water resources (e.g., small streams and pools) often fill in the spring when stream flows are highest, then dry later in the summer. Our observations are that during years with more precipitation these small ephemeral water resources tend to be more abundant and last longer than in drier years. Developing an improved understanding of how bats use the water resources available to them during wet and dry years would lead to a better understanding of how a changing climate might impact different forest-dwelling species.

Future research and population modeling efforts related to bat populations in the Southern Rocky Mountains, and elsewhere in western North America, would benefit from more research in three key areas. First, there is a lack of long-term capture and mark-recapture data related to *Myotis* species in the Southern Rocky Mountains, and elsewhere in western North America. Collecting high quality mark-recapture data, for example, and developing estimates of species-specific survival and fertility rates under various environmental conditions and in different ecoregions would improve substantially on the modeling effort presented here. The multi-year mark-recapture data and analysis related to big brown bats (*Eptesicus fuscus*) associated with the Fort Collins urban area, Colorado [17,45], provides a framework for developing vital rate estimates in natural *Myotis* populations. A multi-decade project similar to the Fort Collins bat project, but conducted in a forested area, would greatly improve our knowledge of how *Myotis* populations respond to varying climate and surface water conditions in the Southern Rocky Mountains.

A second area of research that would improve on the modeling presented here would be to develop capture probability estimates of *Myotis* species related to reproductive status. Adams and Hayes [34] found that reproductive female fringed myotis visited a water resource near a maternity roost significantly more often than non-reproductive females, and presumably more frequently than males, of the same species. This suggests that, because reproductive females visit water resources more frequently than non-reproductive females and males, reproductive females may be more likely than non-reproductive females to be captured in mist nets set at water resources, and thus fertility estimates based on mist-net capture rates that we used in this analysis may be higher than exhibited by the natural population. Robust capture-mark-recapture studies as described above, in addition to estimating vital rates, could be used to help understand variation in capture probabilities among sex, age, and reproductive classes.

A third area of research that would improve our knowledge of forest bat populations in the Southern Rocky Mountains and elsewhere in western North America would be the continuation and development of additional long-term research projects similar to the forest bat monitoring project described by Adams [25] in the Boulder County Front Range. Turchin [49] has emphasized that multi-decade empirical time series data is needed to analyze the population dynamics of terrestrial animal populations. Such long-term capture and monitoring data, along with field experiments [e.g., 34], would complement rigorously collected mark-recapture data and allow a theoretical-empirical synthesis [49] of forest bat population dynamics in the Southern Rocky Mountains and elsewhere in western North America.

An improved understanding of the geographic distributions, use of maternity roost sites, and population trends in forested areas of western North America [26,50–53] and under key climate change scenarios [25,34] will also help biologists and managers protect bat populations in arid regions of western North America. For example, a plausible and testable hypothesis is that fringed myotis (and other forest bat) distributions will tend to erode along the edges of current distributions in areas where fertility and population dynamics models suggest that population growth rates will be lowest (e.g. in southern New Mexico and Arizona for fringed myotis; Fig 3). Given that in this analysis some simulated bat populations decreased to near 0 in just 77 years, biologists, resource managers, and policy makers may need to take proactive actions to support populations and species of concern that are most vulnerable to population erosion under future climate scenarios. In addition to the research discussed above, these actions might include: working with public land management agencies and private land-owners to identify and protect key maternity roost and water resources used by reproductive females and their young; minimizing human disturbance and predation at key maternity roosts, for example by installing bat gates where appropriate; taking action to prevent the spread of bat diseases into key maternity and other roost sites; protecting, and when appropriate, enhancing foraging habitat associated with forest and riparian areas near key maternity roost sites; creating artificial water resources near maternity roosts (e.g., [34]); and minimizing or preventing disruptive human activity, such as recreational rock climbing and noisy forest treatment procedures near maternity sites during the late spring and summer months. All of these conservation tactics would be focused on supporting or improving fertility rates and maintaining high survival rates, and thus help maintain conditions to support stable populations.

Because of their small size and nocturnal habits it is difficult to estimate population sizes of bat species over large spatial scales, such as that of the Southern Rocky Mountain region, or the contiguous United States [19,20]. As a result, there is currently a lack of high quality estimates of population sizes and trends of most North American bat species. Thus, we unequivocally emphasize that the simulation results presented here have only been used as first approximation models [44] to evaluate the conjecture that a changing climate can have non-trivial impacts on some simulated bat populations. Nevertheless, the current lack of reliable population estimates makes conservation and management planning challenging, especially in the face of other recently emerged threats to North American bat populations such as diseases (for example, white-nose syndrome) and wind energy related bat fatalities. Even under optimal conditions, temperate zone insectivorous bat species usually exhibit slow population growth rates due to the tendency of these species to give birth to one young per year and high mortality rates during a bat’s first year of life [17]. Given their contribution to mammalian species diversity in North America and their ecological and economic importance, it will be helpful to continue monitoring and research on the current and future impacts of changing climate conditions on bat populations in western North America. It would also be helpful to support efforts to estimate and monitor bat distributions and populations throughout North America (e.g., North American Bat Monitoring Program [54]), especially if such efforts collect and analyze reliable information on species occurrence, age structure and reproductive rates, and contribute to collecting long-term time series data over multiple decades from repeated sampling of the same study sites.
